# Knowledge, attitudes and practices regarding rabies and its control among dog owners in Kigali city, Rwanda

**DOI:** 10.1371/journal.pone.0210044

**Published:** 2019-08-20

**Authors:** P. Ntampaka, P. N. Nyaga, F. Niragire, J. K. Gathumbi, M. Tukei

**Affiliations:** 1 Department of Veterinary Medicine, University of Rwanda, Nyagatare, Rwanda; 2 Department of Veterinary Pathology, Microbiology and Parasitology, University of Nairobi, Nairobi, Kenya; 3 Department of Applied Statistics, University of Rwanda, Kigali, Rwanda; Universidade Nova de Lisboa Instituto de Higiene e Medicina Tropical, PORTUGAL

## Abstract

**Background:**

Rabies is a zoonotic viral disease that can occur in all warm-blooded animals, including humans. Vaccinating dogs can protect people from contracting rabies. Rabies is a public health threat in Rwanda, but the country does not have information on the epidemiology of rabies. The present study aimed to understand the knowledge, attitudes and practices (KAP) of rabies and its control among dog owners in Kigali city of Rwanda.

**Methods:**

We conducted a cross-sectional survey using a structured questionnaire among 137 dog owners selected from nine administrative study sites. A two-stage random sampling procedure was used to select the participants. Frequency distributions analysis and a series of chi-square tests of associations as well as binary logistic regressions were performed to determine the important factors associated with the response variables.

**Results:**

The results showed that 99.5% of respondents knew at least a host susceptible to rabies. Only 22.4% and 21.3% knew that dogs and people can develop rabies, respectively. Nearly 73.6% knew that human rabies can be transmitted through dog-bites and 99% could identify at least a clinical sign of canine rabies. Overall, 81.8% knew that regular vaccination of dogs helps to prevent dog-transmitted human rabies and 43.1% and 26.3% were aware that rabies in humans and in dogs are fatal once clinical symptoms have shown, respectively. Only 69% would observe a dog for 10 days after it bites a man or an animal. Approximately 20.4% were familiar with appropriate cleaning of dog-bites wounds, and 20.6% knew that puppies could receive rabies vaccination before they are three months old. Of those who owned vaccinated dogs, 78% were happy about the cost (US $ ≤ 34) of rabies vaccination. Of all the respondents, 58% had their dogs vaccinated at home by veterinarians while 86% indicated their veterinarians kept rabies vaccines on ice in a cool box. Overall, 53% of the dog owners had sufficient knowledge of rabies, whilst 66% and 17% adopted adequate practices and positive attitudes towards rabies, respectively. Multivariable logistic regression analyses indicated that none of the respondents’ sex, educational level, and the length of dog ownership were statistically associated with their knowledge, attitudes and practices of rabies.

**Conclusions:**

This study showed that majority of the dog owners had sufficient knowledge and adopted appropriate practices of rabies. However there exist some knowledge gaps among the dog owners particularly on treatment, transmission and control methods. Therefore, rabies awareness campaign is required to upgrade rabies knowledge of the dog owners on rabies prevention and control in Rwanda.

## Introduction

Rabies is a zoonotic viral disease caused by Lyssavirus. Rabies can occur in all warm-blooded animals including humans [1]. Rabies is mainly transmitted through dog-bites in developing countries and causes an approximately 59,000 human deaths worldwide and 8.6 billion USD economic loss annually [2]. In Africa, an estimated 21,476 human deaths occur each year due to dog-mediated rabies (36.4% of global human deaths) [3]. Rabies can be prevented through dog vaccination whilst human rabies deaths can be prevented by washing the bite wound with soap and water, administration of post-exposure rabies vaccine and infiltration of rabies immunoglobulin around the bite wound [3].

Rabies is a serious public health problem in Rwanda [4]. Between January and August 2016, 413 cases of dog-bites in humans were reported across Rwanda and one person reportedly died of rabies [5]. In Rwanda, majority of dogs are guard, although few are kept as pets or for business. Dog-bites in humans are predominantly caused by un-confined and stray dogs. Rwanda Agriculture and Animal Resources Development Board (RAB) is involved in the prevention and control of animal diseases including rabies. Rabies control in Rwanda is implemented through vaccination of owned dogs and culling of stray dogs annually [6]. Annual campaigns for rabies vaccination in Rwanda are communicated through radio news [7].

A report by RAB indicated that, in 2016, there were an estimated 18,117 dogs with 11,375 dogs being vaccinated against rabies (62.7% coverage) and 2,870 dogs culled [8]. People at risk of contracting rabies (veterinarians, medical and national parks staff) do not receive pre-exposure vaccination. Post-exposure first aid (bite wound washing with soap and water and antiseptic application to the bite wound) to dog-bites victims is given at health centers before referring them to the hospitals [7]. The dogs that bite people and animals are rarely quarantined and wildlife species are also not vaccinated against rabies [9].

In Rwanda, fluorescent antibody test (FAT) and RT-PCR tests are used by Rwanda national veterinary laboratory to confirm animal rabies [10], but poor collaboration between veterinary and medical personnel negatively impacts on the surveillance and control as well as on the awareness of the public on rabies [9]. Considering that there are 6 stages that endemic countries for dog-transmitted rabies must go through in order to eliminate the disease [11], Rwanda is reported to be at stage 0 in 2017 [12]. Countries with no information on rabies start at stage 0, while others may start further along the scale, and when the country reaches stage 5, it is free from dog-transmitted rabies [11]. This means that rabies is likely to be present in Rwanda, but the country does not have information on the epidemiology of rabies.

Understanding the community and dog owners knowledge, attitudes and practices (KAP) of rabies, dog-bites in humans and dog management is important because of their influence on post-exposure treatment-seeking behavior and because community support is essential for a rabies prevention and control program [13,14]. Although several KAP studies were conducted elsewhere [13,15,16,17] that provided critical information on rabies, to our understanding, no studies were conducted on KAP of rabies among the community of Kigali city of Rwanda. In this study, we conducted a questionnaire survey among the dog owners of Kigali city, Rwanda. The study findings are expected to provide information that can be used to improve rabies prevention and control in dogs and in humans through targeted community-based education program.

## Methods

### Study area

**Fig 1:**
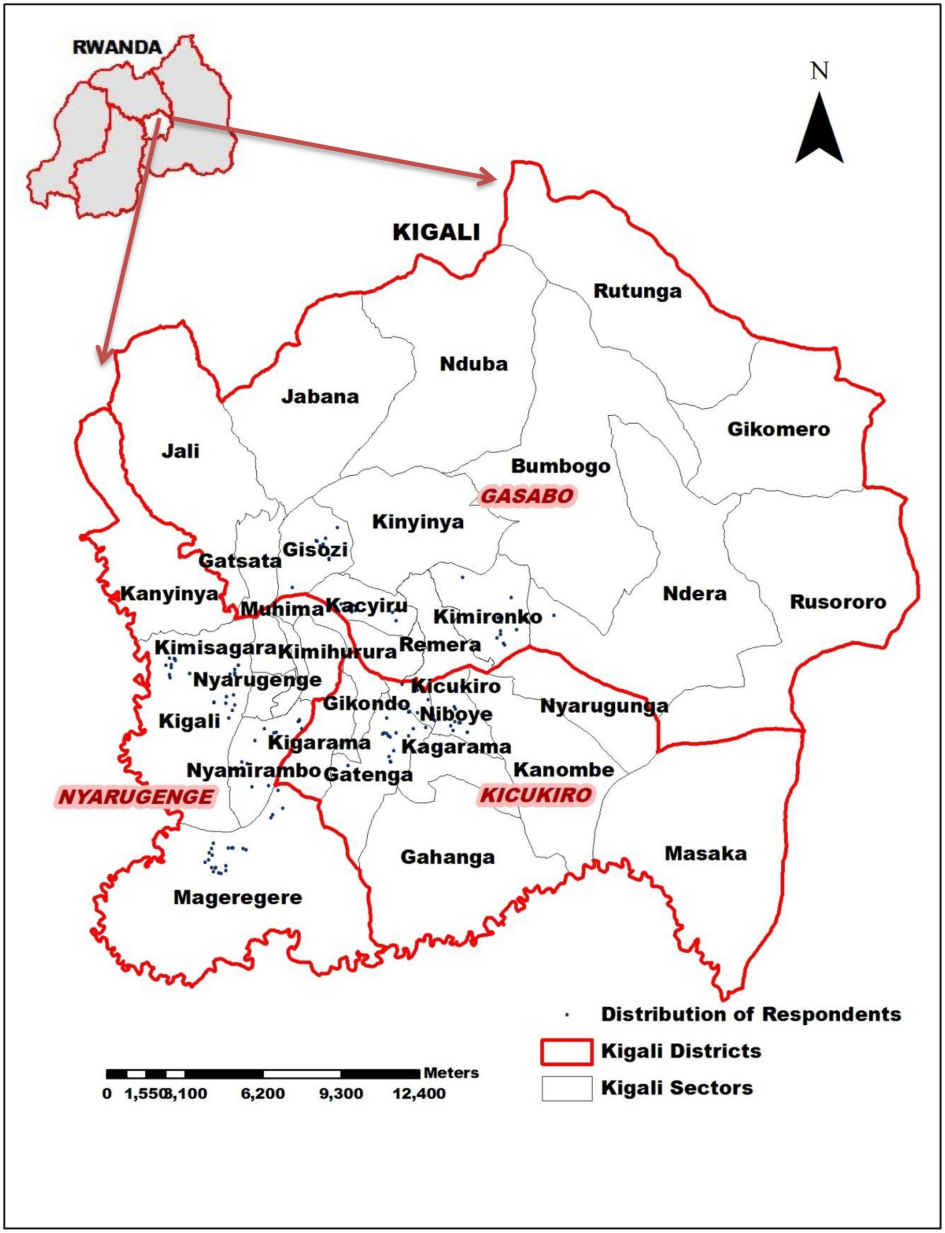
“Fig 1” illustrates names of the three administrative districts of Kigali city which are coloured in red, while those of its administrative sectors are coloured in gray. The blue points represent the respondent homes. The nine study sectors are Mageragere, Nyamirambo and Kigali in Nyarugenge district; Gatenga, Niboye and Kicukiro in Kicukiro district, and Kacyiru, Kimironko and Gisozi in Gasabo district. During this study, GPS data were collected on the location of each respondent’s household. ArcGis10.2 software was used to produce the map based on the GPS data.

The present study was carried out in Kigali, the capital city of Rwanda from September 2016 to February 2017. Rwanda is a landlocked country located in Eastern Africa and Kigali city is one of its 5 provincial administrative entities. Each provincial entity is subdivided into districts, which in turn are subdivided into sectors [18]. Kigali city is divided into three districts, namely, Nyarugenge, Gasabo and Kicukiro (Fig 1).

### Study design

This study adopted a cross-sectional prospective design. It involved data collection from a selected sample of dog owners living in Kigali city through face-to-face interviews that were conducted using a structured questionnaire. Kigali city was purposively selected based on the fact that it was home to a large proportion of dog population in Rwanda. In 2016, records from the three district-level registers indicated a dog population of 2,157 accounting for 12% of the total number of dogs (18,117) in Rwanda.

By the time of the present study, the population of dog owners was unknown because a household can own more than one dog. In absence of no prior report about the level of knowledge, attitudes or practices related to rabies in Rwanda, a theoretical proportion of 50% was assumed [19,20].

Allowing a margin of error (confidence interval or degree of accuracy) of 10% and a 95% level of confidence, the minimum sample size (*n*) of dog owners required for this study was calculated using Cochran’s sample size formula for categorical data [21] as

$$N=\frac{\left(Z_{\alpha ⁄2}^{2}\right)\left(0.5\right)\left(0.5\right)}{{\left(0.1\right)}^{2}}=\frac{{\left(1.96\right)}^{2}\left(0.25\right)}{0.01}=96$$

where *Z*_*α*/2_ = 1.96 is the quantile of the standard normal distribution for a two-tailed test with α = 0.05 level of significance corresponding to 95% level of confidence. The fact that people living in cities are employed in time-demanding jobs including business and public services resulted in having trouble finding the respondents at their homes. For these reasons, we decided to increase the calculated minimum sample by 50% to compensate for potential non-response [21,19]. Thus this study targeted a sample of 144 dog owners, of whom interviews were successfully conducted among 137 dog owners. The present study followed a two-stage random sampling procedure in order to select the participants. In the first stage, a simple random sampling was applied to select three administrative sectors from each of the three districts of Kigali city to form a sample of nine administrative sectors. In the second stage, information from district-level registers on dog vaccination against rabies was used to construct a sampling frame for each of the nine sectors, under the assumption that each household owned at least one dog. In case a household owned many dogs, one interview was conducted in each selected household.

With probability proportional to each sector’s dog population size, a systematic random sampling technique was utilized in order to select the households that participated in this study.

### Questionnaire interview

A structured questionnaire was designed to collect the data from the respondents. A questionnaire was pretested before the actual field survey. Through collaboration with local administrative authorities, dog owners were contacted by phone or in person. The selected respondents, either head of a household or one adult member (>18 years of age) were interviewed. The respondents were briefed about the purpose of the study and an informed written consent was obtained prior to the interview. The questionnaires were administered in their local language (Kinyarwanda) by the interviewer and their answers were recorded in English.

### Ethical considerations

The Rwanda National Ethics Committee reviewed and approved this study (Review Approval Notice: No. 15/RNEC/2017).

### Data management and analysis

During the interview, each respondent was given an identifying unique code that linked the respondents to their individual characteristics and responses during the data analysis process.

Data organization and analysis were carried out using International Business Machines (IBM) Statistical Package for Social Sciences (SPSS) Statistics for Windows, version 20. In addition to frequency distributions analysis, tests of associations of knowledge, attitudes and practices regarding rabies with the respondent’s individual characteristics, namely educational level, sex, district of residence and length of dog ownership were carried out using chi-square tests of associations.

Further, an index was constructed using principal components factor analysis (PCFA) for each of the three components of interest, namely, the level of knowledge, attitudes and practices towards rabies based on the respondent’s responses to corresponding questions [22]. For data preparation for PCFA, items that were used to measure the three dimensions were transformed into indicator variables. First, the six items that were used to measure knowledge of rabies aimed to investigate whether the respondents knew: (i) *hosts who can suffer from rabies;* (ii) *how rabies can be transmitted between dogs and other animals*; (iii) *clinical signs of canine rabies*; (iv) *the prognosis for clinical canine rabies*; (v) the *most effective method for controlling canine rabies* (vi) *how best dog-mediated rabies can be controlled in humans*.

This study chose a set of items used to constitute a parsimonious indicator of the respondent’s level with respect to each of the three dimensions, namely the knowledge, attitudes and practices regarding rabies. The respondent who inadequately answered any of the items was considered having inadequate level of relevant dimension. This study selected all the six items to collaboratively constitute a minimum indicator of sufficient knowledge of rabies among the respondents with special focus on key issues related to susceptibility to rabies, mode of transmission, clinical description and prevention and control of rabies. Thus, any respondents who have not correctly answered these six items were categorized as having insufficient knowledge of the disease. The same consideration applied to the other two dimensions namely the attitudes and practices of rabies. Respondents’ knowledge was classified as either *sufficient*, if they could provide correct answers to all the six items or *insufficient* otherwise. The respondents’ attitudes towards rabies were measured using two items that aimed to find out: (i) *what they would wish to happen to the biting dog if it is caught;* and (ii) *what they can do before taking a colleague who is bitten by a dog to a health care facility*.

The attitude was considered as either *positive*, if the respondent indicated a correct attitude for the two items, or negative otherwise. The respondents’ practices regarding rabies were assessed by investigating: (i) *how often the respondents took their dog(s) to vaccination*; (ii) *how a veterinarian who previously vaccinated the respondent’s dog(s) carried and administered the vaccine*. The practices were classified as either *appropriate*, if the respondent adopted good practices vis-à-vis these items, or *inappropriate* practices otherwise. All the indicators of the respondent’s knowledge, attitudes or practices regarding rabies were categorized into two and coded with 1 for sufficient knowledge, positive attitudes, and appropriate practices or coded with 0 otherwise.

A binary logistic regression analyses were conducted for each of outcome variables, namely, knowledge, attitudes, and practices regarding rabies to understand the associations of each outcome variable with the respondents’ characteristics [23,17]. In the first instance, each of the three outcome variables associations with each of the respondents’ characteristics were assessed using Pearson chi-square test. In the second place, all the respondents’ characteristics with p-values < 0.3 for at least one of the outcome variables were considered for multivariable logistic regression analyses. Any variables with P-value ≤0.05 were considered significant in the final model. The model goodness-of-fit was assessed by the Hosmer-Lemeshow test [17].

## Results

### Demographic characteristics of the respondents

Overall, 137 respondents were interviewed including 107 (78.1%) owning dogs that received rabies vaccination. Male respondents represented 65.7% of the total sample. Of all the respondents, 37.2% completed or were still undertaking tertiary education, while 30.7% and 28.5% finished primary and secondary school, respectively. Approximately 3.6% did not get formal education. Among the respondents, 33.6% and 22.6% had owned dogs for > 10 and for 5–10 years, respectively, whilst 43.8% had kept dogs for < 5 years.

The respondents who lived in Nyarugenge district accounted for 41.5% while those from Kicukiro and Gasabo districts represented 34.5% and 24%, respectively (Table 1).

**Table 1:** Distribution of the sample of households across selected administrative sectors and districts in Kigali city, Rwanda.

Districts	Sectors	Frequency	Percentage
Gasabo	Gisozi	10	7
	Kacyiru	12	9
	Kimironko	11	8
Nyarugenge	Nyamirambo	17	12.5
	Kigali	15	11
	Mageragere	25	18
Kicukiro	Niboye	20	15
	Gatenga	21	15
	Kicukiro	6	4.5
Total		137	100

#### Respondents’ knowledge on rabies

Sixty seven percent of the respondents were aware of rabies in humans, dogs, cats and jackals while 32.5% knew that farm animals (cows, sheep, goats, pigs and rabbits) can get rabies. Approximately 0.5% did not have an idea on susceptibility of various hosts to rabies (Table 2).

**Table 2:** Respondents’ knowledge on susceptible hosts of rabies, transmission modes and clinical signs of rabies in dogs.

Variables	Counts (n = 137)	Cases percentage	Valid percentage
**Respondents’ knowledge on susceptible hosts of rabies**			
Dogs	133	97.1	22.4
Cats	73	53.3	12.3
Cows	44	32.1	7.5
Sheep	39	28.5	6.6
Goats	41	29.9	6.9
Pigs	37	27.0	6.2
Rabbits	31	22.6	5.2
People	126	92.0	21.3
Jackals	66	48.2	11.1
Do not know	3	2.2	0.5
**Total**	593*^a^		100**
**Respondents’ knowledge on mode of animal rabies transmission**			
Bites	120	87.6	65.2
Wound licking	29	21.2	15.8
Skin scratching	8	5.8	4.4
Food	12	8.8	6.5
Intact skin licking	1	0.7	0.5
Coitus	2	1.5	1.1
Inhalation of aerosolized saliva	2	1.5	1.1
Do not know	10	7.3	5.4
**Total**	184*^a^		100**
**Respondents’ knowledge on clinical signs of rabies in dogs**			
Aggressiveness	112	81.8	27
Profuse salivation	85	62.0	20
Eating abnormal items	43	31.4	10
Difficulty in swallowing	7	5.1	2
Roaming over long distances	97	70.8	23
Change in sound	31	22.6	7
Dropping of the jaw	41	29.9	10
Do not know	2	1.5	1
**Total**	418*^a^		100**

* Percent of positive responses

** Actual percentage

Overall, 85.4% of respondents correctly knew how rabies can be transmitted between dogs and other animals (bites, licking of wounds and skin scratches), while 9.2% did not understand the rabies transmission modes since they mentioned that it can be transmitted through food, licking intact skin, coitus, and inhalation. The rest (5.4%) of respondents did not have an idea on transmission of animal rabies. Regarding the knowledge of transmission of human rabies, 73.6%, 16.5% and 7.7% of the respondents were aware of bites, licking of wounds and skin scratches as modes of transmission, respectively while 2.2% indicated wrong route of transmission (Table 2).

Seventy percent of the respondents knew that rabid dogs are aggressive, roam aimlessly and salivate profusely. Twenty nine percent were familiar with eating non-edible substances, dropping of the jaw, hoarse sound, and difficult swallowing. One percent indicated they would not be able to recognize a rabid dog based on clinical signs (Table 2). The study showed that 42.3% of the respondents believed that human clinical rabies can be treated successfully. Nearly 43.1% understood that it is always fatal, while 14.6% did not know the fate of human rabies. In addition, 54% of the respondents believed that a dog showing clinical signs of rabies can be treated successfully. Also, 26.3% knew the fatal nature of canine rabies while 19.7% did not understand the fate of treatment of canine rabies.

Regarding the knowledge of rabies prevention, 81.8% of the respondents considered regular vaccination of dogs as a method of choice for protecting people from getting rabies, while 18.2% believed that other methods would be enough to prevent and control human dog-mediated rabies. These methods included killing of stray dogs (5.9%), education of the public (5.1%), complete restriction of dogs (2.9%), immunization of people at risk of developing rabies (2.9%), and treatment of people after the exposure (1.4%).

### Sources of information about rabies

The respondents have heard about rabies from various sources with majority being from friends and neighbors (39%), the media (29.9%) and the public meeting (13.1%) (Table 3).

**Table 3:** Sources of information about rabies for the respondents.

Sources of information	Counts (n = 137)	Cases percentage	Valid percentage
Friends or neighbours	98	71.5	39.0
The media	75	54.7	29.9
Public meetings	33	24.1	13.1
Parents	18	13.1	7.2
Schooling	13	9.5	5.2
Veterinarians	12	8.8	4.8
At work	2	1.5	0.8
Total	251*a1		100**

* Percent of positive responses

** Actual percentage

### The respondents’ attitudes towards unprovoked human dog bites

When asked about what would they do with the dogs that bite people or animals, 69% revealed that they would keep a dog for 10 days observation once it bites a man or an animal to verify whether it is rabid or not, irrespective of the dog’s vaccination status. The percentage of respondents who would kill or release the dog immediately accounted for 15.5% for each category, regardless of dogs’ vaccination and ownership status. When asked about caring for a dog bite patient, 68.6% of the respondents indicated they would take the patient to a hospital before they do anything. The results showed that 20.4% of the participants would clean the victim’s wounds with water or with both water and soap depending on its availability; while 8% would dress the bite and put a bandage. Those who would put salt on the wound or clean it with 70% alcohol or povidone-iodine represented 1.5% each.

### The respondents’ practices on rabies control

We found that only 20.6% of the respondents who owned vaccinated dogs (n = 107), knew that puppies younger than three months old can receive rabies vaccination and had vaccinated theirs when they were of such age. The dogs were vaccinated by the veterinarians at their homes (57.9%) or at vaccination points during vaccination (41.2%). Less than 1% took their dogs to veterinary clinics for rabies vaccination. In addition, the dog owners indicated that, at time of vaccination, vaccines were transported into the field using ice packs in a cool box (86%) or in a plastic bag (12.1%). Our results showed that 78% of the respondents were happy with the cost of rabies vaccination, which varied up to the Rwandan Franc (RWF) 30000 (1 US $ is equivalent to 878 RWF). Those respondents who owned unvaccinated dogs (n = 30) indicated that the main reasons for not vaccinating their dogs included lack of information (39.6%), negligence on their part (37.2%), inadequate knowledge of rabies (11.6%), due to vaccination fees (9.3%), and vaccination points located far from their homes (2.3%).

### Overall status of knowledge, attitudes and practices towards rabies among dog owners

The factor scores obtained from PCFA for each participant and for each of the (i) knowledge (ii) attitudes and (iii) practices towards rabies were ranked in descending order and grouped into two levels with all cases below the 50^th^ percentile (high factor scores) was ranked in the first level and the latter groups included the dog owners who had sufficient knowledge, positive attitudes or appropriate practices. Table 4 shows the frequency distribution of the dog owners according to the constructed indicators. The results indicate that on average, 53% of the dog owners had sufficient knowledge of rabies, whilst 66% and 17% adopted adequate practices and positive attitudes towards rabies, respectively.

**Table 4:** Knowledge, attitudes and practices towards rabies among dog owners in Kigali City (n = 137).

Dimension	Level	Dog owners	Percent
Knowledge	Sufficient	73	53.3
	Insufficient	64	46.7
Attitudes	Positive	24	17.5
	Negative	113	82.5
Practices	Adequate	91	66.4
	Inadequate	46	33.6

### Logistic regression analyses

The analysis of associations of each of the outcome variables with respondents’ characteristics is presented in Table 5. The results showed that each of the factors considered in this study, namely, sex, educational level, district of residence and length of dog ownership, had a p-value less than 0.3 for at least one of the outcome variables. We decided to consider all the four factors for the subsequent multivariable analyses.

**Table 5:** Test of associations (*χ*^2^) between the respondents’ knowledge, attitudes and practices towards rabies in Kigali, Rwanda (n = 137).

	Knowledge		Attitudes		Practices	
Factors	Sufficient (%*)	P-value	Positive (%*)	P-value	Adequate (%*)	P-value
***Sex***						
Male	43 (47.8)		18 (20.0)		64(71.1)	
Female	30(63.8)	0.074	6 (12.8)	0.290	27 (57.4)	0.108
**Educational level**						
Tertiary	29 (56.9)	0.660	10 (19.6)	0.956	30 (58.8)	0.496
Secondary	22 (56.4)		6(15.4)		28 (71.8)	
Primary	19 (45.2)		7(16.7)		30 (71.4)	
No formal education	3 (60.0)		1(20.0)		3(60.0)	
**District of residence**						
Nyarugenge	25 (43.9)		12 (21.1)		42(73.7)	
Kicukiro	26 (55.3)		6 (12.8)		26 (55.3)	
Gasabo	22 (66.7)	0.106	6 (18.2)	0.539	23(69.7)	0.128
**Length of dog ownership**						
>10 years	24 (52.2)	0.742	12 (26.1)	0.172	29(63.0)	0.569
5–10 years	15 (48.4)		4 (12.9)		23 (74.2)	
< 5 years	34 (56.7)		8 (13.3)		39 (65.0)	

* Percent of the respondents who had sufficient knowledge and adopted positive attitudes or adequate practices within each level of the considered factor

About 64% and 57% of female respondents had sufficient knowledge and adopted appropriate practices, respectively. Few of male (20%) and female (12.8%) respondents adopted positive attitudes towards rabies. The attitude was generally negative across all categories of the four factors considered in this study. The results revealed that the length of dog ownership does not significantly improve the dog owner’s knowledge nor the practices regarding rabies. Majority of the respondents living in Nyarugenge district (73.7%) adopted adequate practices whilst most of those who lived in Gasabo district (66.7%) had sufficient knowledge of rabies. None of the predictor variables, namely respondent’s sex, educational level, district of residence or the length of dog ownership was statistically associated with the respondent’s knowledge of rabies. Similarly, there was no statistically significant association of the status of dog owners’ attitudes or practices regarding rabies with any of the selected predictor variables. However, there were some considerably different relationships between the status of the respondents’ knowledge, attitudes and practices and the length of dog ownership (Table 6).

**Table 6:** Multivariable logistic regression analysis of factors of knowledge, attitudes and practices towards rabies in Kigali city.

		Knowledge	Attitudes	Practices
Variable	Category	OR (95% CI)	OR (95% CI)	OR (95% CI)
District	Gasabo	*1*.*00*	*1*.*00*	*1*.*00*
	*Kicukiro*	0.59(0.23,1.54)	0.65(0.18,2.33)	0.55(0.21, 1.44)
	*Nyarugenge*	0.42(0.15,1.18)	1.74(0.46,6.51)	0.98(0.32, 2.98)
Sex	*Female*	*1*.*00*	*1*.*00*	*1*.*00)*
	*Male*	0.60(0.27,1.34)	1.47(0.49,4.42)	1.40(0.62, 3.13)
Educational level	*Tertiary*	*1*.*00*	*1*.*00*	*1*.*00*
	*Secondary*	1.24 (0.51,3.05)	0.59(0.18,1.97)	1.51(0.59,3.86)
	*Primary*	0.97 (0.37,2.54)	0.50(0.14,1.85)	1.42 (0.52,3.88)
	*No education*	2.12(0.27,16.45)	0.41(0.03,5.40)	0.71 (0.09, 5.66)
Length of dog ownership	*> 10 years*	*1*.*00*	*1*.*00*	*1*.*00*
	*5–10 years*	0.96 (0.37, 2.50)	0.35(.10, 1.26)	1.46(0.52,4.13)
	*< 5 years*	1.23 (0.54, 2.79)	0.39(.14, 1.11)	0.97(0.42,2.27)
Goodness-of-fit test		*	**	***

*Hosmer-Lemeshow goodness of fit test statistic: 10.333, P- value = 0.242

** Hosmer-Lemeshow goodness of fit test statistic: 3.289, P-value = 0.915

*** Hosmer-Lemeshow goodness of fit test statistic: 7.946, P-value = 0.439.

Specifically, the odds of having sufficient knowledge of rabies for the respondents residing in Nyarugenge district were about 60% lower than they were for the respondents residing in Gasabo district. The odds of having sufficient knowledge of rabies were 2.12 times higher for those who kept dogs for < 5 years than they were for those who owned dogs for ˃ 10 years. Similarly, the odds of having sufficient knowledge of rabies were 1.23 times higher for those with no formal education than they were for those with tertiary education.

In addition, the odds of adopting adequate practices towards rabies among the respondents who completed primary and secondary education were respectively 1.51 and 1.42 times higher than they were among those who finished or were undertaking tertiary education.

Furthermore, the odds of adopting positive attitudes towards rabies among the respondents who resided in Nyarugenge district were 1.47 times higher than they were among those who lived in Gasabo district. The odds of taking positive attitudes towards rabies were at least 60% lower for any length of dog ownership than they were for those who owned dogs for ˃ 10 years. Similarly, the odds of adopting positive attitudes were at least 40% lower for the respondents with any educational level than they were for the respondents with tertiary education. The assessment of models fit to data with Hosmer-Lemeshow goodness of fit test showed that all the three models were good fit to data, with all P-values greater than 0.05.

## Discussion

This study aimed to understand the knowledge, attitudes and practices of rabies and its control among dog owners in Kigali city, Rwanda. To our understanding, this is the first KAP study on rabies conducted among the community of Kigali city of Rwanda.

Our findings are expected to guide decision makers to improve rabies prevention and control in dogs and in humans through targeted community-based education program. The majority of the respondents had sufficient knowledge and adopted adequate practices of rabies whilst < 20% took positive attitudes towards rabies. Almost all the respondents were aware of human or animal rabies and majority of them knew the proper ways (bites, licking of wounds and skin scratches) through which humans and animals can contract rabies. Nearly 73.6% of the respondents knew that human rabies can be transmitted through dog-bites and this was consistent with previous studies by Fenelon et *al*. [24] in Haiti and by Tiwari *et al*.[17] in India. We found that 9.2% of the respondents lacked understanding on transmission of animal rabies. Similarly, inconsistencies in rabies knowledge were reported in previous studies conducted in Ethiopia: intact skin [25] and inhalation [25,26].

Although there is no documented evidence so far that rabies had been transmitted through sexual intercourse with rabid patients, rabies transmission through sexual intercourse with rabid patients is of course possible if there is violent sex with bite and kiss (bite kiss). Although 99% of our respondents could identify at least a clinical sign of canine rabies, only 43% and 26% knew that clinical human and canine rabies are almost always fatal, respectively. Similar reports by Sambo *et al*. [15] in Tanzania and Matibag *et al*.[13] in Sri Lanka found that majority of the respondents (63–78.8%) were aware of fatal nature of clinical human rabies. Considering that, once rabies manifests clinically, it cannot be treated successfully [27], it is very clear that majority of our respondents had serious lack of knowledge of rabies treatment. To improve rabies knowledge among the respondents, awareness needs to be strengthened and channelled through radio and television broadcast and community meetings.

The improvement of knowledge of canine rabies in a community can be achieved via a structured awareness campaign [28]. Although majority (81.8%) of our respondents considered regular vaccination of dogs to be the method of choice for preventing human rabies, only 20.6% of those who owned vaccinated dogs had vaccinated their puppies against rabies before they were 3 months old. This could indicate that the majority did not know ages at which dogs could receive rabies vaccination for the first time. World Health Organization [29] advises dog owners and vaccination teams to consider vaccinating puppies including newborns as young dogs constitute a large proportion of dog population in majority of countries endemic for rabies. The rabies incidences are high in puppies born from an unvaccinated dam. Rabies transmission from puppies to children is high since children would play with dogs particularly puppies. So there is higher risk of human beings contracting rabies from young puppies [30, 31, 32].

Our findings revealed that the main sources of rabies information among the respondents were from neighbours, radio and television and public meetings. Similar reports found neighbours [33] and families [16] to be the leading sources of rabies information for the respondents in Ethiopia whilst Ghosh *et al*. [34] found radio and television to account for 30% of respondents’ sources of rabies information in Bangladesh. Therefore, it is important that rabies awareness education in Kigali city be conducted using mass media such as radio and television broadcast.

Only 20.4% of our respondents were aware of the proper first aid to dog-bites victims. Similar studies by Sambo *et al*. [15] in Tanzania and by Dhiman *et al*. [35] in India reported comparable findings, that is 5% and 43.07%, respectively. The small number of the respondents who knew about first aid and the fact that some of them would cover the patient’s wound with dressings and bandages or put salt on the wound indicated lack of knowledge of dealing with wounds of dog-bites. It is not allowed to apply irritants (e.g. chilli powder, plant juices, acids or alkalis) to an animal bite wound or to use dressings or bandages to cover the wound [36]. Human rabies deaths can be prevented by washing the bite wound with soap and water, administration of post-exposure rabies vaccine and infiltration of rabies immunoglobulin around the bite wound [3]. This study revealed that some of the respondents (31%) would kill or release a dog once it bites an animal or a man regardless of the dog’s vaccination and ownership status. In contrast, such a dog is supposed to be quarantined and observed [37] for 10 days to exclude rabies [38].

Our findings disagreed with those of other authors [39,40] who found that respondents’ knowledge of rabies was influenced by their educational level. The fact that all the respondents owned dogs and that the prime source of rabies information was neighbours could indicate that dog owners interacted to share knowledge of rabies regardless of their educational level. Our findings were also incompatible with those of Mucheru *et al*. [41] in Kenya who revealed that owning a dog for a long time for the respondents could make them realise the benefits of vaccinating their dogs regularly. Our study however agreed with that of da Costa and Fernandes [40] in Brazil who found that sex did not influence knowledge of rabies among respondents.

Like any other observational studies, our study has some limitations. Due to difficulties in finding the respondents, we were unable to interview the expected number of the respondents. Furthermore, the fact that sampling framework was constructed based on information from district-level registers on dog vaccination, this could have led to some bias.

## Conclusion

This study showed that majority of the dog owners had sufficient knowledge and adopted appropriate practices of rabies.

However there exist some knowledge gaps among the dog owners particularly on treatment, transmission and control methods. Therefore, rabies awareness campaign is required to upgrade rabies knowledge of the dog owners on rabies prevention and control in Rwanda.

## Supporting information

S1 File Survey questionnaire. (PDF)

S2 File Certificate of consent for the respondents. (PDF)
