# Supplementary material for: Knowledge, attitudes and practices regarding rabies and its control among dog owners in Kigali city, Rwanda
Source: PLoS One. 2019 Aug 20;14(8):e0210044. doi: 10.1371/journal.pone.0210044 (PMC6701806; doi:10.1371/journal.pone.0210044)
Supplement: S1 File — (PDF) [file pone.0210044.s001.pdf]

Survey questionnaire

Interview date:…......./………../2016

Tel. number: …………………………………….

Dog owner ID: Code:……...

i) Dog owner particulars

1.District…………………Sector……………….....Cell……….....………Village……………….

2. GPS reading: (a) land mark number :……………(b) Longitude…………………

(c) Latitude…………………… (d) Elevations……………………………

3. Sex: (a) Male ☐ (b) Female ☐

4. Educational level: (a) No formal education, ☐ (b) Primary, ☐ (c) Secondary, ☐ (d) Tertiary ☐

ii) Dog keeping

5. How long have you been keeping dog (s)? *Tick one*

(a) < 5 years ☐ (b) 5-10 years ☐ (c) >10 years ☐

6. Why do you keep dog (s)? *Tick one*

(a) Security ☐ (b) pet ☐ (c) herding ☐ (d) accidentally ☐

iii) Knowledge of rabies

7. Have you heard about rabies disease? (a) Yes ☐ b) No ☐

8. If yes, how did you get information about rabies disease? *Tick as appropriate*

(a) On the radio ☐ (b) Reading of hard or online newspapers, books, etc ☐ (c) Public meeting ☐

(d) Neighbours ☐ (e) Parents ☐ (f) veterinarians ☐ g) schooling ☐ (h) Other, ☐ specify

9. Do you know susceptible hosts to rabies? (a) Yes ☐ (b) No ☐

10. Which of the following hosts can suffer from rabies? *Tick appropriately*

(a) Dogs ☐ (b) cats ☐ (c) cows ☐ (d) sheep ☐ (e) goat ☐ (f) pigs ☐ (g) rabbits ☐
(h) People ☐ (i) Jackal ☐ (j) other, ☐ specify….

11. How rabies can be transmitted between dogs and other animals? *Tick as appropriate*

(a) Bite ☐ (b) licking of wound ☐ (c) skin scratch ☐ (d) food ☐ (e) licking of intact skin ☐ (f) other, ☐ specify,

12. How rabies can be transmitted from dogs to humans? *Tick as appropriate*

(a) Bite ☐ (b) licking of wound ☐ (c) licking of intact skin ☐ (d) skin scratch ☐

(e) Do not know ☐

13. Do you know clinical signs of rabies in dogs? (a) Yes ☐ (b) No ☐

14. Which of the following clinical signs are seen in dogs with rabies? *Tick as appropriate*

(a) Aggressiveness (biting without any provocation) ☐ (b) Profuse salivation ☐ (c) Pica (e.g., sticks, nails, faeces, etc) ☐ (d) Difficulty in swallowing ☐ (e) Roaming over long distances (running for no apparent reason) ☐ (f) Change in sound (e.g., hoarse barking or inability to make sound) ☐ (g) Dropping of the jaw ☐ (h) Other, ☐ specify (i) do not know ☐

15. What is the prognosis for rabies in dogs showing clinical signs? *Tick one*

(a) They can be treated successfully ☐ (b) they always die ☐ (c) do not know ☐

16. What is the prognosis for dog mediated rabies in people showing clinical signs? *Tick one*

(a) They can be treated successfully ☐ ,(b) they always die ☐ , (c) do not know ☐

17. What is the most effective method for rabies control in dogs? *Tick one*

(a) Killing of stray dogs ☐ (b) restriction of dog movements ☐ (c) regular vaccination

(d) Castration ☐

(iv) Attitudes towards rabies

18. If a dog bites a man or an animal, what would you wish to happen to the biting dog if is caught? *Tick one*

(a) Immediate release if the owner is known and the dog is vaccinated ☐

(b) Direct killing if the owner and vaccination status are unknown ☐

(c) Keep the dog for 10 days to see if it is rabid; regardless of its vaccination status ☐

19. If your colleague is bitten by a dog, what can you do before you take him/her to a health care facility? *Tick one*

(a) Careful wash of the wound with water alone or with soap if available ☐ (b) Covering the wound with dressings and bandages (c) Apply the salt to the wound ☐ (d) Take the patient to a health care without doing anything ☐ (e) Apply 70 % alcohol to the wound ☐ (f) apply other type antiseptics to the wound ☐ specify…..

20. In your thinking, how best dog-mediated rabies can be controlled in humans? *Tick one*

(a) Regular vaccination of dogs ☐ (b) Complete restriction of dogs ☐ (c) Education of the public ☐ (d) Killing stray dogs ☐ (e) regular vaccination of people at risk (e.g., veterinarians)

(f) Post-exposure prophylaxis ☐

(v) Practices of rabies

21. Do you bring your dog (s) to vaccination against rabies? (a) Yes ☐ b) No ☐

22. How old was your dog at first vaccination?

(a) Younger than or three month old ☐ (b) older than three months ☐(c) do not know ☐

23. How do you proceed to have your dog (s) vaccinated? *Tick one*

(a) Take a dog to a site during campaign for vaccination, ☐ (b) a veterinarian comes at home ☐ ,(c) both approaches ☐ ,(d) take a dog to a veterinary clinic ☐

24. Who vaccinates your dog (s)? *Tick one*

(a) Private veterinarians ☐ (b) public veterinarians ☐

25. How a veterinarian who vaccinated your dog last time did carry the vaccine? *Tick one*

(a) In a cool box ☐ (b) on ice without cool box (e.g., in a plastic bag) ☐

(c) The vaccine was purchased and carried on ice by "the dog owner" and then administered by a veterinarian ☐

26. How much did you pay to have your dog vaccinated last time? *Tick one*

(a) 1- 1, 500 FRW ☐ (b) 1,501-3,000 FRW ☐ (c) 3,001 – 5,000 FRW ☐ (d) 5,001 - 10,000 FRW ☐ (e) 10,001 – 20,000 FRW ☐ (f) 20,001-30,000 FRW ☐) free of charge ☐

27. What do you think of the vaccination fees? *Tick one*

(a) Too little money ☐ (b) too much money ☐ (c) affordable ☐

28. Why have you not taken your dog (s) to vaccination? "Owners of unvaccinated dogs"- Tick *as appropriate*

(a) Lack of information ☐ (b) lack of knowledge of rabies ☐ (c) difficulty in catching dogs ☐ (d) too much vaccination fees ☐ (e) Sites set far during campaigns for vaccination ☐ (f) Negligence ☐

Thank you for your cooperation!!!!
