# Supplementary material for: Knowledge, attitudes and practices regarding rabies and its control among dog owners in Kigali city, Rwanda
Source: PLoS One. 2019 Aug 20;14(8):e0210044. doi: 10.1371/journal.pone.0210044 (PMC6701806; doi:10.1371/journal.pone.0210044)
Supplement: S2 File — (PDF) [file pone.0210044.s002.pdf]

Certificate of consent for respondents

I have been explained about the present research and have had the opportunity to ask questions and any questions that I have asked have been answered to my satisfaction. I consent willingly to take part as a participant in this research.

Name of Participant: …………………………………………………………………

Signature of Participant : ………………………………………………………………..

Date:………/………../201…

If illiterate

I have witnessed the precise explanation of the consent form to the potential participant, and the individual has had the opportunity to ask questions. I confirm that the individual has given consent freely.

Name of witness: ……………………………………and thumb print of participant

Signature of witness:………………………………………..

Date………/………/201..

Statement by the principal investigator

I have accurately explained about the research to the potential participant, and to the best of my ability made sure that the participant understands that interviews will be conducted, and that the data will be confidentially kept. I confirm that the participant was given an opportunity to ask questions about the study, and all the questions asked by the participant have been answered properly and to the best of my ability. I confirm that the individual has not been forced into giving consent, and the consent has been given freely and voluntarily.

Name of the principal investigator taking the consent……………………………………..

Signature of the principal investigator taking the consent…………………………………

Date: ………/………/201…
